# Prognosis prediction of stage IV colorectal cancer patients by mRNA transcriptional profile

**DOI:** 10.1002/cam4.4824

**Published:** 2022-05-19

**Authors:** Bian Wu, Jinwei Yang, Zhiwei Qin, Hongping Yang, Jingyi Shao, Yun Shang

**Affiliations:** ^1^ Department of General Surgery II the First People's Hospital of Yunnan Province Kunming Yunnan China; ^2^ Department of General Surgery Wenshan people's Hospital of Yunnan Province Yunnan China; ^3^ Department of Anus and Intestine Surgery Qujing Hospital of Traditional Chinese Medicine Qujing Yunnan China; ^4^ Department of Reproductive Medicine the First People's Hospital of Yunnan Province Kunming Yunnan China

**Keywords:** colorectal cancer, expression, mutation, prognosis, RNA‐seq, transcription, WES

## Abstract

**Background:**

Stage IV colorectal cancer patients with liver metastasis represent a special group of CRC patients with poor prognosis. The prognostic factors have not been investigated for stage IV CRC patients undergoing primary cancer resection but not candidates for metastasis resection.

**Methods:**

Ninety‐nine stage IV CRC patients who underwent primary cancer resection without metastasis resection were retrospectively recruited. Both whole‐exome sequencing (WES) and RNA‐seq were performed with frozen primary cancer tissues, using para‐cancerous normal tissues as the control. Valid data were obtained from 78 patients for WES and 84 patients for RNA‐seq. Univariate, multivariate Cox analyses were performed and Nomogram model was established to predict patient prognosis.

**Results:**

The correlation between patient prognosis and clinicopathological factors, mutational status, or mRNA level changes was examined. Univariate (*p* = 0.0007) and subsequent multivariate analyses on clinicopathological factors showed that location (left or right) was the only independent risk factor for patient prognosis (HR = 3.63; 95% CI: 1.56–8.40, *p = 0*.003), while T, N, M staging, gender, race, location (rectum or colon), and pathological types were not stratifying factors. The mutational status of APC, TP53, KRAS, TTN, SYNE1, SMAD4, PIK3CA, RYR2, and BRAF did not show significant stratification in patient prognosis. RNA‐seq showed that genes related to membrane function, ion channels, transporters, or receptors were among those with significant mRNA level alterations. Univariate analysis identified 97 genes with significantly altered mRNA levels, while NEUROD1, FGF18, SFTA2, PLAC1, SAA2, DSCAML1, and OTOP3 were significant in multivariate analysis. A risk model was established to stratify the prognosis of stage IV CRC patients. A Nomogram model was established with these genes to predict individual patient prognosis.

**Conclusions:**

A panel of eight genes with significant mRNA level alterations was capable of predicting the prognosis and risk of the specific patient group. Future prospective study is needed to validate the model.

## Graphical abstract

This study aimed to clarify the prognostic factors for stage IV CRC patients undergoing primary cancer resection but not liver metastasis resection. WES and RNA‐Seq were used to sequence primary cancer tissues of 78 and 84 patients, respectively. Models were established to stratify the prognosis and to predict individual patient prognosis. This is the first time that an mRNA profile‐based model was established to predict the prognosis for the specific group of patients.

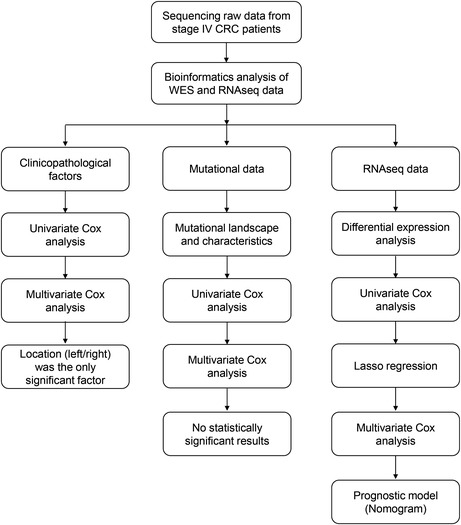

## 1 INTRODUCTION

Colorectal cancer is one of the leading cause of cancer‐related morbidity and mortality worldwide.^1^, ^2^ Patients with stage IV CRC represent a subpopulation of CRC with distant metastasis and serious complications. Approximately 20%–30% of the CRC patients are diagnosed with distant metastasis upon their first medical visit, from which 50% of these cases are affected by liver metastasis.^1^, ^2^ It has been reported that the 5‐year survival rate for stage IV CRC was inferior to 10%.^1^, ^2^ Patients with stage IV liver metastatic CRC can be treated with a series of systematic therapies, combined with surgical procedures. Palliative surgery on primary cancer with postsurgical chemotherapy on liver metastasis becomes a routine method for this type of patients.^3^, ^4^ The goal of the surgery is to remove the tumor tissue, at the highest level, to allow tumor remission, and also increase the possibility of disease maintenance for further chemotherapeutic treatment.^3^, ^4^ Therefore, the purpose of a combined therapy is not to cure the condition but instead for reliving symptoms, prolonging survival, and enhancing life quality. Despite the great advancement on surgical techniques and subsequent systematic therapy during recent years, 2/3 of the patients will experience cancer relapse in a 5‐year period.^3^, ^4^

In spite of the broad utilization of surgery followed by chemotherapy in the treatment of stage IV CRC patients with liver metastasis, the prognostic, and risk factors for these patients are largely unknown. Previous studies mainly focused on the prognosis of all stage IV patients,^5^, ^6^, ^7^ or stage IV patients with no surgery opportunities who underwent systematic chemotherapy, targeted therapy, or immunotherapy,^8^, ^9^, ^10^ or stage IV patients who underwent synchronous or staged radical surgery on both primary cancer and metastasis.^11^, ^12^ Little information is available on the prognosis of stage IV patients who underwent primary cancer surgery without the resection of liver metastasis.^13^ Due to the lack of effective biomarkers, it has not been possible to predict the prognosis or survival of these patients. The relationship between the disease prognosis and clinicopathological, genetic factors, transcriptional, or expression profile has not been fully examined. Meanwhile, an undifferentiated therapeutic regimen may result in unnecessary treatment, with no clear expectation of therapeutic response and/or long‐term survival. In order to clarify the influencing factors involved in the therapeutic response and prognosis of this specific population, we planned to investigate the putative prognostic and risk factors that impact stage IV CRC patients with liver metastasis underwent palliative surgery with subsequent chemotherapy. By recruiting CRC patients with liver metastasis (patients recommended for surgery on primary cancer followed by chemotherapy) retrospectively, the information for genetic alterations and transcriptional alterations of primary cancer tissues was obtained, and the clinicopathological and follow‐up information was collected. We aimed to identify factors that may potentially predict the prognosis of these patients. Specifically, we hoped to reveal the independent risk factors for patient survival and, ultimately, to establish a model for the prediction of individual survival and risk.

## 2 MATERIALS AND METHODS

### 2.1 Ethics, patients, and samples

A retrospective cohort study was designed and implemented at the First People's Hospital of Yunnan Province. This research was approved by the ethics committee of the hospital and conducted in accordance with the hospital's guiding principles (approval number: KHLL2019‐018). Samples were selected, recruited, and tests were performed based on the confirmed diagnosis and the availability of clinical and follow‐up information. Patient information was kept anonymous for confidentiality throughout the process of sample collection and testing.

Frozen samples collected from 2008 to 2020 were recruited in this study. Tissue samples of primary cancer were collected from surgery, and were snap‐frozen in liquid nitrogen and moved to −80 °C freezer for long‐term storage. The inclusion criteria included adults over 18 years old, patients with complete clinicopathological information and follow‐up information, and a confirmed diagnosis of CRC with liver metastasis (including endoscopy, MRI, or CT), and subsequent pathological examinations. All samples were confirmed to be colorectal adenocarcinoma by pathological examinations. Patients were included for those who received surgery on primary cancer but were not candidates for liver metastasis resection (i.e., patients received systematic therapy on liver metastasis following surgery on primary cancer). The availability of primary cancer tissue samples was a requirement for inclusion. The exclusion criteria included those patients not treated by surgery with subsequent chemotherapy, a history of cancers other than CRC, unavailability of samples or follow‐up information. Patients with cancer types other than adenocarcinoma were also excluded. Thus, a cohort of 99 patients with confirmed CRC with liver metastasis were available for study. In the subsequent WES, valid data were obtained from 78 patients, who passed the sample quality control, library control, sequencing control, and data control. In the subsequent RNA‐seq, valid data were obtained from 84 patients who passed the above controls. Para‐cancerous histologically normal tissues were also collected and sequenced as the control.

### 2.2 Methods for whole‐exome sequencing (WES)

DNA from frozen tissue samples was extracted using the EasyPure ® Genomic DNA Kit (Beijing TransGen Biotech). The quality control for the DNA was achieved using Qubit 2.0 (Thermo Fisher Scientific), following the manufacturer's instructions. The fragmented genomic DNA underwent end‐repairing, A‐tailing, and ligation, and then was sequentially completed with indexed adapters, followed by size selection using Agencourt AMPure XP beads (Beckman Coulter Inc.). Library construction was performed with the DNA fragments using the KAPA Library Preparation kit (Kapa Biosystems, Inc.) according to the manufacturer's protocol. Seven to eight polymerase chain reaction (PCR) cycles, depending on the amount of DNA used, were performed on pre‐capture ligation‐mediated PCR (Pre‐LM‐PCR) oligos (Kapa Biosystems, Inc.) in 50 μL reactions. DNA sequencing was performed using a WESPlus gene panel (an upgraded version of the standard whole‐exome sequencing (WES), HaploX Biotechnology) for tumor tissue sequencing on the Illumina Novaseq 6000 system according to the manufacturer's instructions. Sequencing data were filtered by Fastp and aligned to the hg19 genome (GRch37) using Burrows Wheeler Aligner (BWA). SAMtools was used to sort the BAM files and perform duplicate marking. The Gencore version 0.12.0 (https://github.com/OpenGene/gencore) was used to remove duplicate reads. Somatic variants were determined using MuTect2. New panel of normal (PON) created by in‐house healthy individual using GATK. ANNOVAR was performed to annotate the Variant Call Format file obtained in the previous step.

### 2.3 Methods for RNA‐seq

RNA from fresh tissue samples was extracted using EasyPure® RNA Kit (Beijing TransGen Biotech, Beijing, China). RNA was quantified with the Qubit 2.0 Fluorometer and the Qubit RNA HS assay kit (Thermo Fisher Scientific, Inc.) according to the manufacturer's instructions. After ribosomal RNA (rRNA) was digested by the Illumina TruSeq Stranded Total RNA Library Preparation Kit (Illumina), the rRNA‐removed RNA was cut into small fragments for cDNA synthesis. Libraries were generated using the KAPA Stranded RNA‐seq Library Preparation kit (Kapa Biosystems, Inc.) according to the manufacturer's protocol. Sequencing was conducted with the Illumina Novaseq 6000 platform. Data processing for differential RNA expression analysis between tumor tissues and normal tissues was performed using the edgeR in R package. Expression with |LogFC| > 2.5 (FC stands for fold change) and adjusted *p* value (false discovery rate control, FDR) < 0.01 were considered to be significant differential expression. The integrated dysregulated gene lists were saved for subsequent analysis. Heatmaps were plotted to show the first 100 genes with differential mRNA level change, and enrichment analysis was conducted based on genes with significant mRNA level change.

### 2.4 Data analysis and model establishment

**FIGURE 1:**
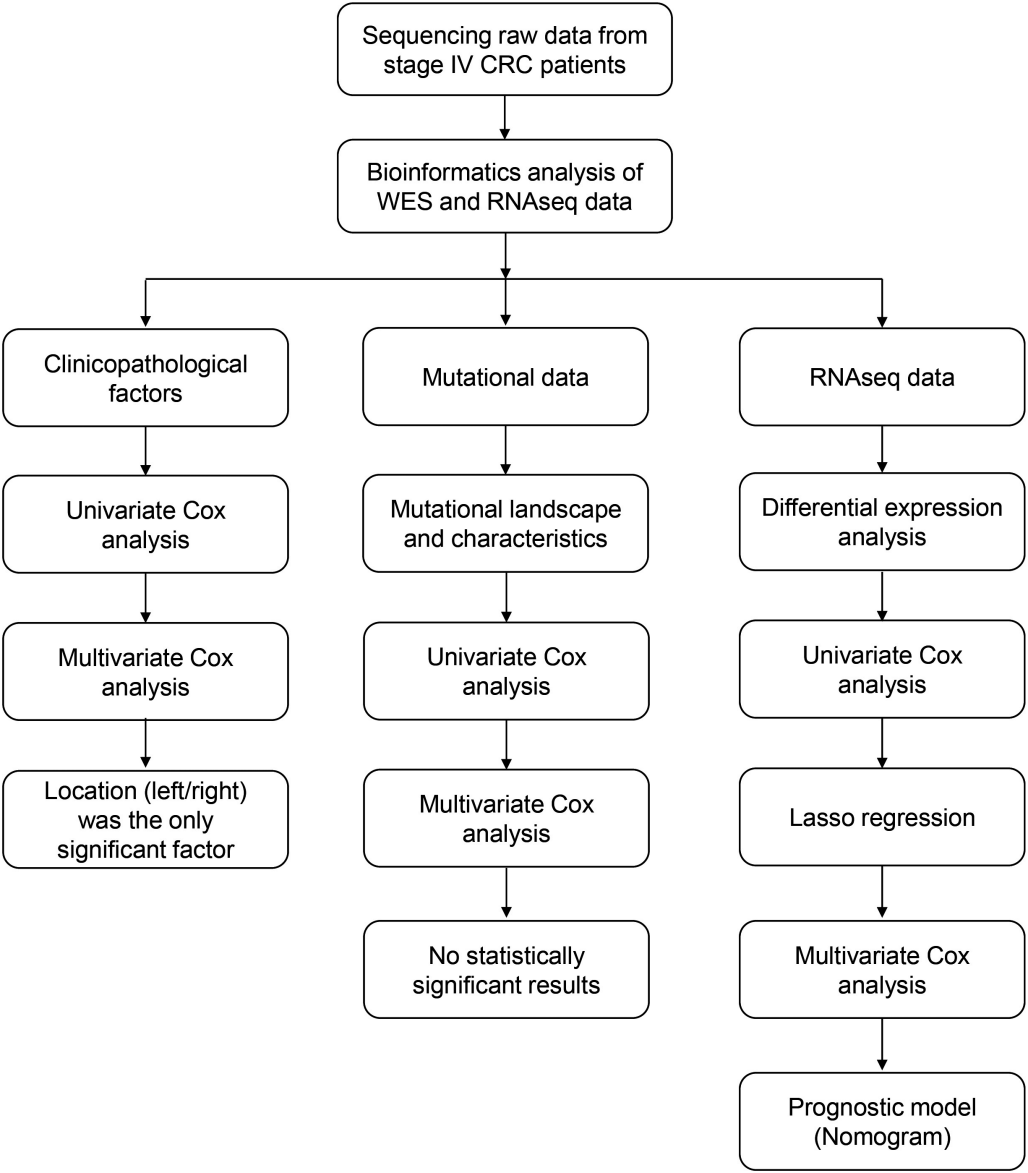
Flowchart for data analysis and model establishment in this study. Both sequencing data in WES, RNA‐seq, and clinicopathological data from patients were collected. Location (left/right) was found to be the only significant risk factor among all clinicopathological factors. Univariate and multivariate Cox analyses were performed with mutational data, along with the prognosis data, while the results revealed no significant mutations for prognosis stratification. In contrast, RNA‐seq data reveal several significant genes in multivariate Cox analysis and a prognostic model (Nomogram model) was established based on the findings.

**FIGURE 2:**
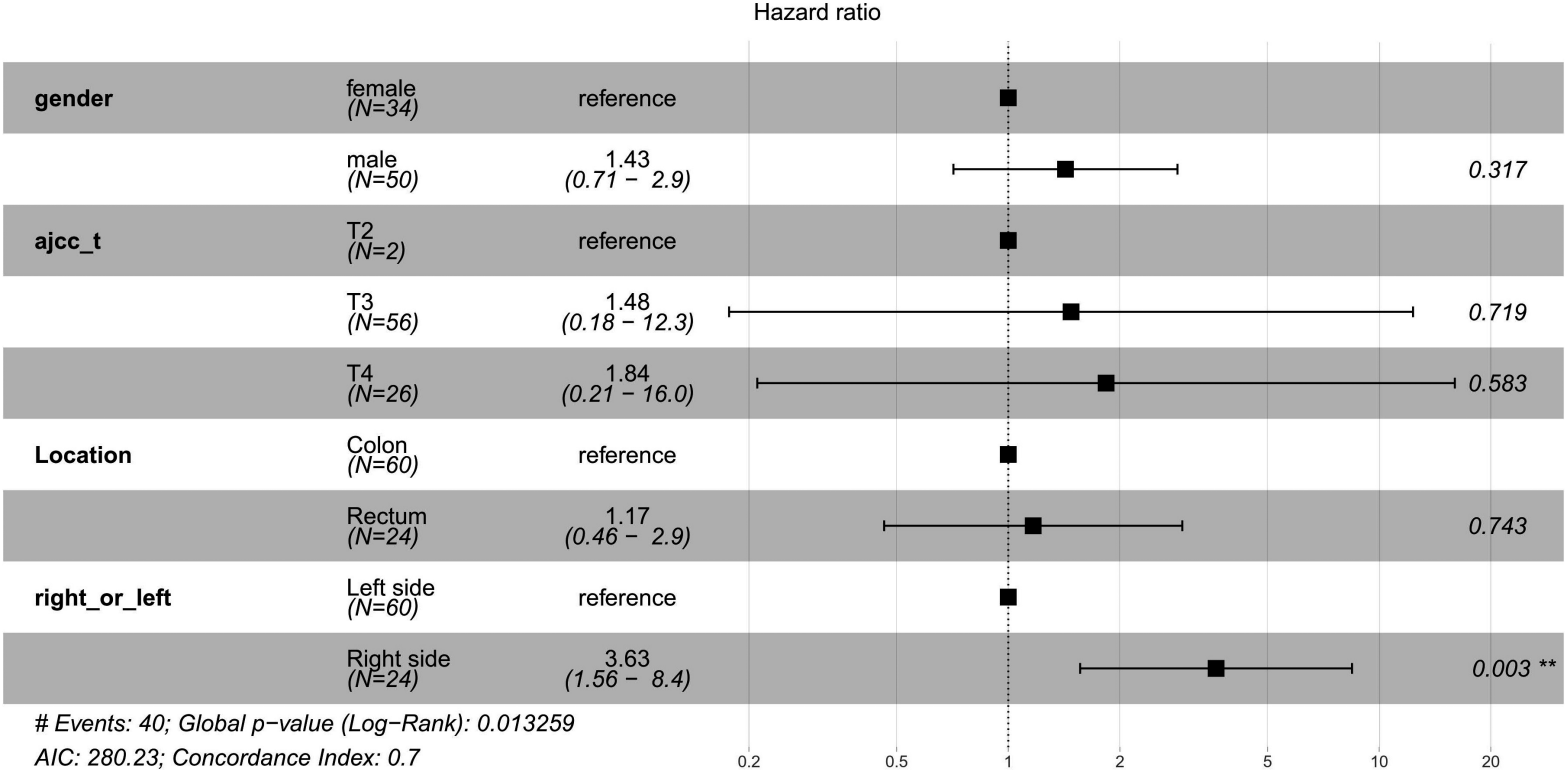
Results for multivariate analysis of clinicopathological factors in this study. Location (left/right) was found to be the independent risk factors for patient prognosis (*p =* 0.003).

**FIGURE 3:**
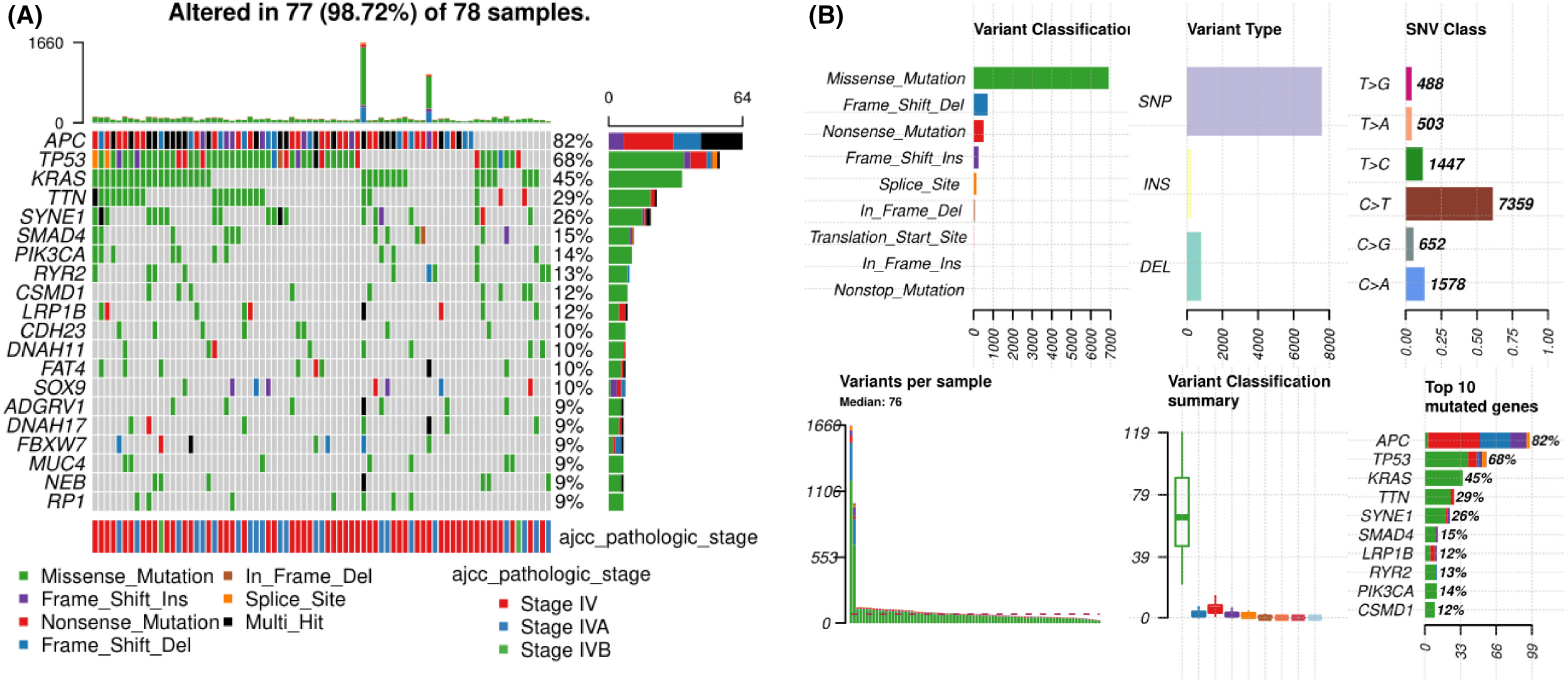
Mutational landscape and characteristics of the primary cancer tissue of stage IV CRC patients in this study. (A) The mutational landscape of 78 patients with valid sequencing data for analysis. APC, TP53, and KRAS were among the genes with highest mutational rate. Mutation types and pathological stages are labeled by colors as indicated. (B) The mutational characteristics, including the variant classification, types, base changes, variants per sample, and top mutated genes are shown in individual panels.

**FIGURE 4:**
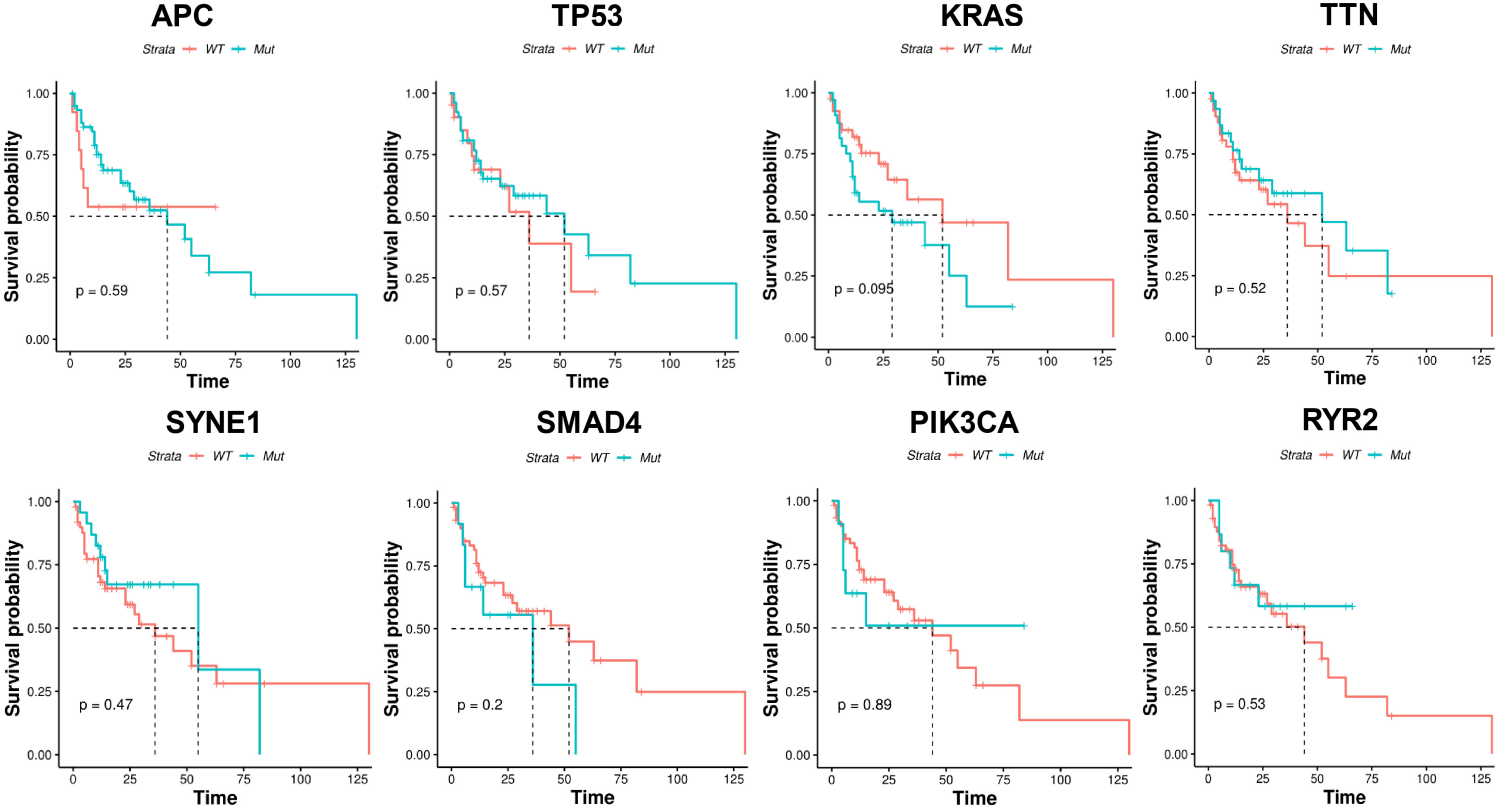
Kaplan–Meier analysis by mutational status. The patient survival was stratified by mutational status (wild type or mutation) of top mutated genes, as indicated in each panel. No significant stratification of prognosis was found among these genes, as indicated by the P values, although the stratification by KRAS was close to significant (*p* = 0.095).

**FIGURE 5:**
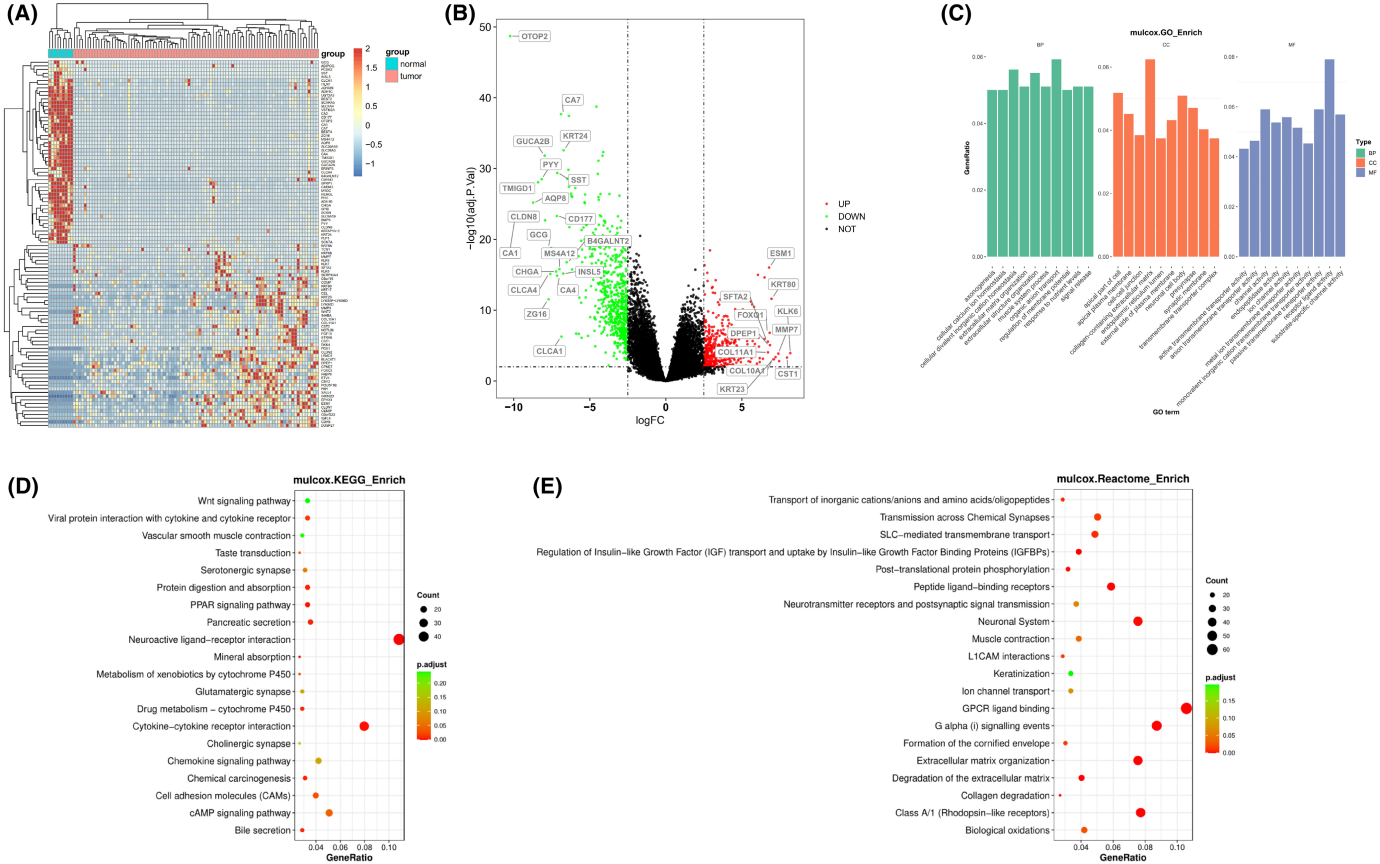
Results for differential mRNA levels between primary cancer tissue and adjacent normal tissues in stage IV cancer patients in this study. (A) heatmap shows the mRNA level difference of the top 100 differential expressed mRNA genes. Blue bar indicates normal control while red bar indicates cancer tissue. (B) Volcano plot shows the significant upregulated and downregulated genes with mRNA level change. *p* < 0.05 and |logFC| > 2.5 were used as thresholds for interpreting significant mRNA level change. (C, D, and E) the results for GO, KEGG, and Reactome enrichment analysis for differentially expressed genes.

**FIGURE 6:**
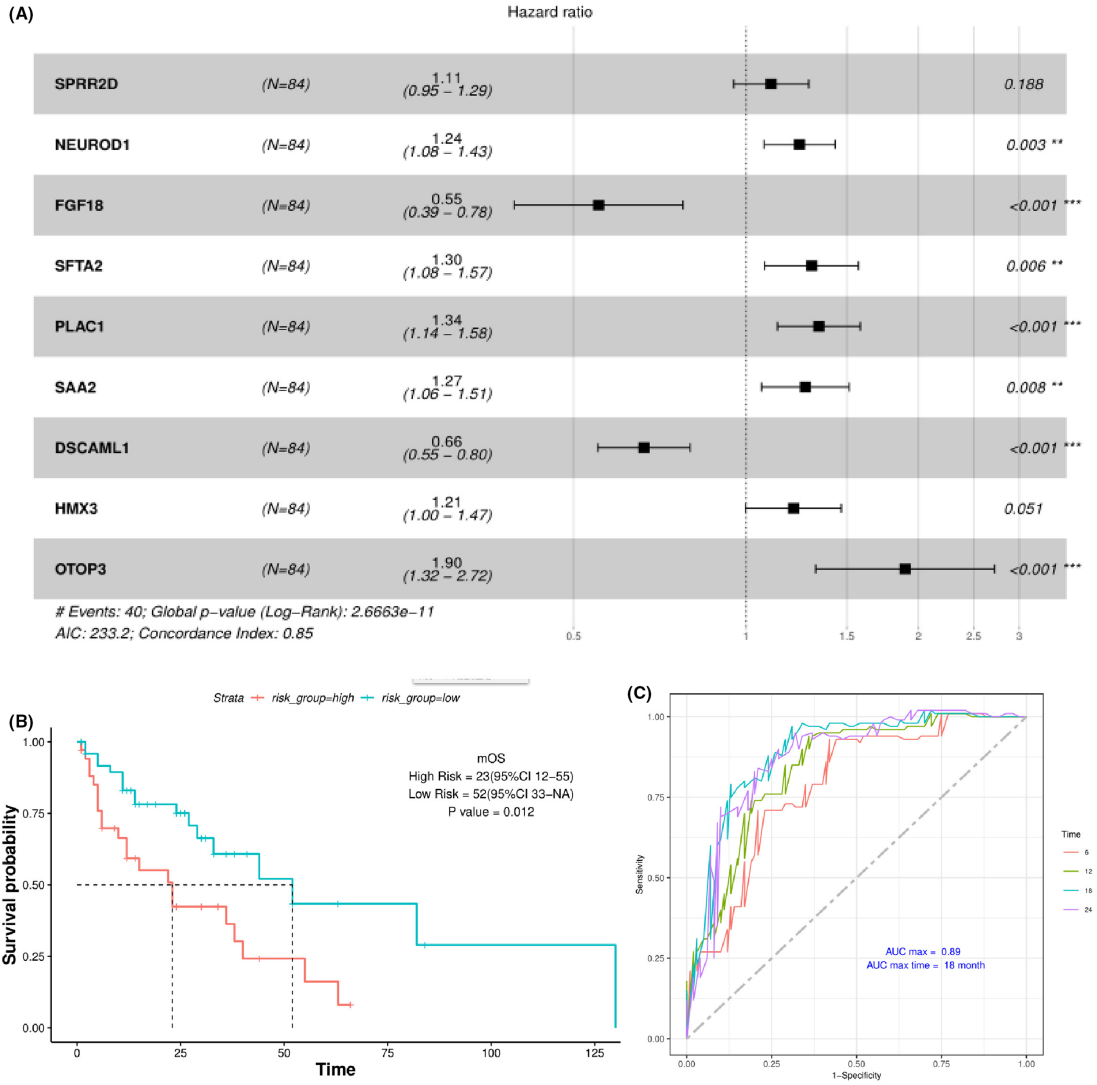
Multivariate analysis and establishment of risk stratification models for the prognosis of stage IV CRC patients. (A) The results of multivariate analysis of genes with differential mRNA levels. Seven genes were found to be significant in the analysis, while the other two also had significant influence on the final risk model and there were also included. (B) Risk stratification and Kaplan–Meier analysis based on results from multivariate analyses. Significant stratification of patient prognosis was found between high‐ and low‐risk patients (*p =* 0.012). (C) The diagnostic model (ROC curves) is shown for the risk stratification model at different time points. Maximal AUC of 0.89 was achieved at 18 months, as indicated.

**FIGURE 7:**
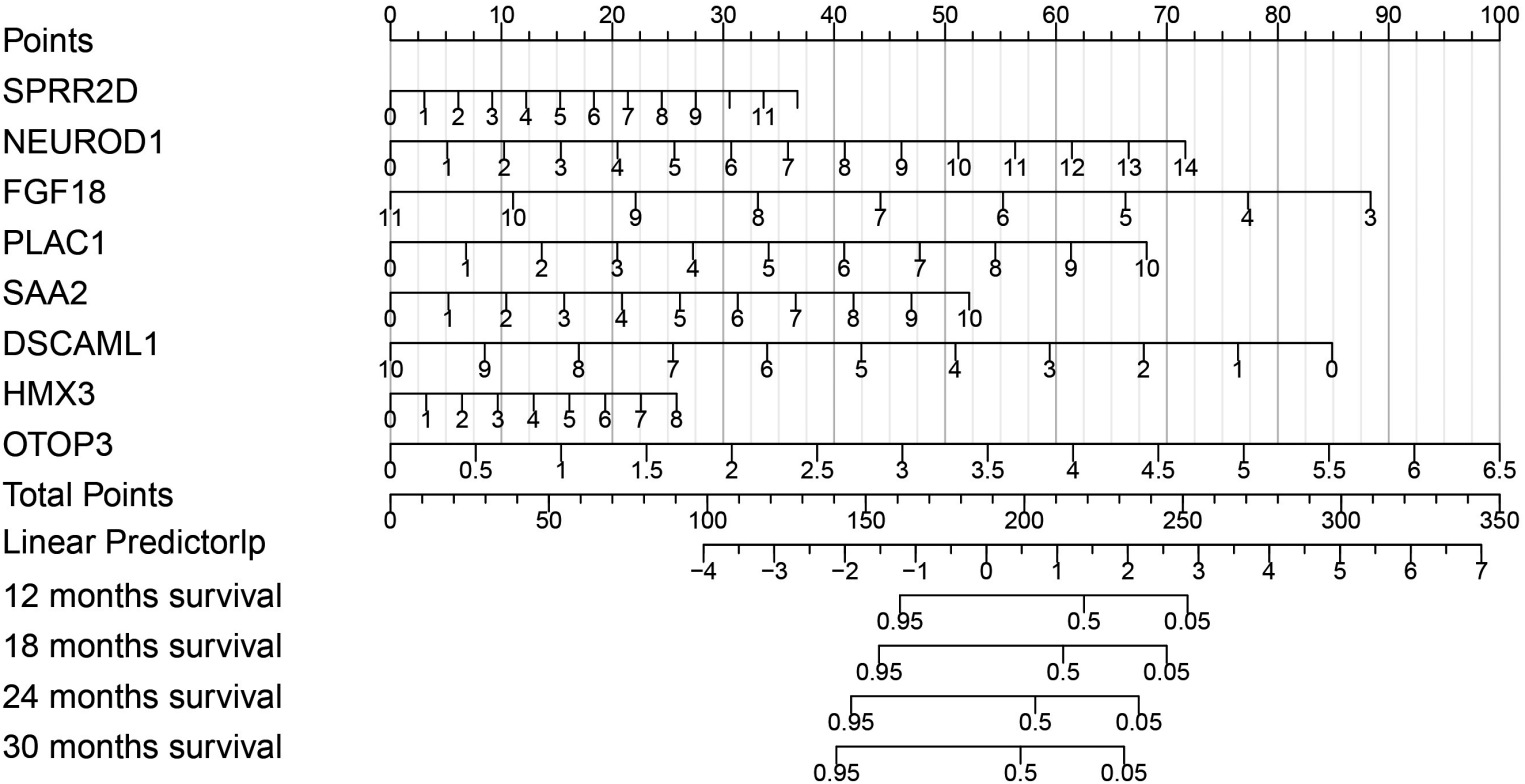
Nomogram model for prediction of the individual risk of stage IV CRC. Genes from the multivariate analysis were used to establish the model. Scores can be calculate for each gene based on mRNA profile, and risk and can be calculated based on the formulation in the result section. Risk can then be quantified by the overall risk score (Linear predictor). The corresponding survival probability at each time point can then be calculated.

The flowchart of in Figure 1 elaborates the data analysis process of this study. The relationship between patient prognosis and clinicopathological factors, mutational status or mRNA levels was analyzed by univariate and multivariate analyses, and P values were corrected by Benjamini & Hochberg (BH) method. For the differentially transcripted genes revealed by RNA‐seq, variable selection was carried out using the “GLmnet” LASSO (Least Absolute Shrinkage and Selection Operator) regression algorithm in R software (Figure 5). Candidate genes were selected for multivariate Cox regression analysis and line‐drawing. Risk score was calculated by the covariate coefficients obtained by Cox regression, and the calculation formula was as follows: E ^sum (each gene's expression × corresponding coefficient). The “maftools” package of the R software was used for plotting the mutational landscape and characteristics (Figure 3). The “pheatmap”, “ggplot2”, and “clusterProfiler” packages of the R software were used for plotting heatmap, volcano plot and the results for GO, KEGG and Reactome enrichment analysis, respectively (Figure 5). R software was used for univariate and multivariate analyses (Table 2; Table SS1; Table SS2), and “forestplot” package was used for plotting the figures for univariate and multivariate analyses (Figure 2, Figure 6A). The “survivalROC” package was used to draw ROC curve and calculate the area under the curve (AUC) (Figure 6C). Patients were divided into high‐risk and low‐risk groups according to the risk score at the maximal difference between true positive and false positive of the maximal AUC (Figure 6C). Survival analysis was conducted with the Kaplan–Meier method and subsequent log‐rank test. Survival curves were plotted using the “survival” and “survminer” package of the R software (Figure 4 and Figure 6B). The Nomogram model was plotted with the “rms” package of the R software (Figure 7).

## 3 RESULTS

### 3.1 The mutational status of main driver genes could not predict the prognosis of stage IV CRC patients

A summary of the clinicopathological information for all patients is listed in Table 1, including patient gender, age, cancer T/N/M stage and clinical stage, cancer location and pathological types. The potential stratification of overall survival time by patient clinicopathologicial factors was first investigated. Univariate analysis (Table 2) shows that location (left or right) was the only significant stratifying factor for patient overall survival time (*p =* 0.0007), while T staging, N staging, M staging, gender, race, location (rectum or colon), and pathological types were not stratifying factors for this specific population (*p* > 0.05 as indicated in Table 2). Further multivariate analysis (Figure 2) shows that location (left or right) was the only independent risk factor for patient overall survival (HR = 3.63; 95% CI: 1.56–8.40, *p =* 0.003), in which patient with right‐side colonic cancer exhibited significantly worse overall survival.

**TABLE 1:** Clinicopathological factors of patients in this study

Clinicopathological factors	Categories	Number (%)
Gender	Female	40 (40.4)
	Male	59 (59.6)
Age	<40	2 (2.0)
	40–49	13 (13.1)
	50–59	16 (16.2)
	60–69	24 (24.3)
	70–79	23 (23.3)
	≥80	14 (14.1)
T stage	T2	2 (2.0)
	T3	63 (63.6)
	T4	34 (34.4)
N stage	N0	12 (12.1)
	N1	38 (38.4)
	N2	49 (49.5)
M stage	M1a	74 (74.7)
	M1b	25 (25.3)
Clinical stage	Stage IVA	74 (74.7)
	Stage IVB	25 (25.3)
Location	Cecum	14 (14.1)
	Ascending	13 (13.1)
	Transversal	2 (2.0)
	Descending	3 (3.0)
	Sigmoid	22 (22.2)
	Rectum	27 (27.3)
	Not specified	18 (18.2)
Pathological types	Tubular adenocarcinoma	87 (87.9)
	Mucinous adenocarcinoma	12 (12.1)

**TABLE 2:** Results of univariate analysis of clinicopathological factors

	HR	95%CI	*p* value
**T staging**			
T2	1	N/A	N/A
T3	1.1	0.15–8.6	0.89
T4	2.8	0.35–22	0.34
T4a	1.4	0.15–13	0.78
T4b	2.2	0.14–37	0.58
**N staging**			
N0	1	N/A	N/A
N1	0.64	0.22–1.8	0.41
N1a	0.42	0.05–3.6	0.43
N1b	9.20E‐09	0‐Inf	1
N1c	9.10E‐09	0‐Inf	1
N2	0.88	0.32–2.4	0.81
N2a	9.20E‐09	0‐Inf	1
N2b	0.47	0.091–2.4	0.36
**M staging**			
M1 all	1	N/A	N/A
M1a	0.66	0.2–2.2	0.5
M1b	1.4	0.19–11	0.72
**Gender**			
Female	1	N/A	N/A
Male	1.1	0.58–2.2	0.75
**Location**			
Colon	1	N/A	N/A
Rectum	0.71	0.33–1.5	0.38
**Location**			
Left	1	N/A	N/A
Right	3	1.6–5.8	0.0007***

The mutational landscape of 78 stage IV CRC patients was then established and the mutational features were characterized. It can be observed from Figure 3A that APC, TP53, KRAS, TTN, and SYNE1 were among the top mutated genes in stage IV CRC patients. Missense mutation was the predominant mutation type, followed by frameshift and nonsense mutation types (Figure 3B). Frameshift and nonsense were the predominant types for APC gene while missense was the main type for other genes (Figure 3B). These observations showed no difference to previous reports on mutational landscape of CRC.

The potential stratification of overall survival time by mutational status was examined next. It can be seen from Figure 4 that the mutational status (mutant or wild type [WT]) of the top mutated genes, including most main driver genes, such as APC (*p =* 0.59), TP53 (*p =* 0.57), KRAS (*p =* 0.095), TTN (*p =* 0.52), SYNE1 (*p =* 0.47), SMAD4 (*p =* 0.20), PIK3CA (*p =* 0.89), RYR2 (*p =* 0.53), and BRAF (*p =* 0.83), showed no significant stratification on patient overall survival time. KRAS showed a trend of stratification, in which patient with no KRAS mutations (WT) exhibited potentially better survival. Univariate analysis was performed to identify genes with stratification potential, and 502 genes were found to be significant in the analysis (Table SS1). Multivariate analysis was performed subsequently, while none of the genes were found to be statistically significant, suggesting that the mutational status of these genes was correlated with other factors and was not independent risk factors for this specific population of stage IV CRC.

### 3.2 The cancer tissue transcriptional change was capable of predicting the prognosis of stage IV CRC patients

The transcriptional alterations of 84 stage IV CRC patients were characterized, and the profile of differential expression profile between cancer tissue and adjacent normal tissue was established by RNA‐seq. Expression profile of the top 100 differentially expressed genes is shown in Figure 5A, and huge difference in expression between normal (blue bar) and cancer tissue (pink bar) can be observed. The full profile of transcriptional changes between normal and cancer tissue is shown by volcano plot in Figure 5B, in which both upregulation and downregulation are demonstrated as indicated. Significant upregulation (red dots) or downregulation of gene expression (green dots) was defined by the *p* value (0.01) and the log FC (fold change) value (absolute value of 2.5). Gene names with the most significant changes between normal and cancer tissue are labeled as indicated. GO, KEGG, and Reactome enrichment analyses were performed with the significant upregulated or downregulated genes (Figure 5 C, D, E). It appeared that genes related to membrane function, ion channels, transporters or receptors were among those with significant enrichment, suggesting that the expression of various membrane proteins were significantly altered in stage IV CRC.

Univariate analysis was performed to identify potential stratifying gene expression for patient overall survival, and 97 genes were found to be significant in the analysis (Table SS2). Subsequent multivariate analysis showed seven significant genes, including NEUROD1, FGF18, SFTA2, PLAC1, SAA2, DSCAML1, and OTOP3 (Figure 6A), and two extra genes (SPRR2D and HMX3) with substantial influence on area under the curve (AUC). Corresponding HR and P values are shown in Figure 6A as indicated. The high‐risk population was distinguished from the low‐risk population in stage IV CRC patients based on the risk score at the maximal difference between true positive and false positive of the maximal AUC (Figure 6B, C). Stratification of patient overall survival using the above model is shown in Figure 6B. It can be seen that patients in low‐risk group exhibited significantly better survival than those in the high‐risk group (*p* < .012), with the median OS at 52 months (95% CI: 33‐NA) and 23 months (95% CI: 12–55), respectively (Figure 6B). The ROC curve was plotted according to the model (Figure 6C). Survival time points at 12, 18, 24, 30, 36 months were tested for model performance, and it was found that the maximal AUC of 0.89 was achieved at 18 months, suggesting the optimal time point for prediction.

In order to facilitate the prediction of patient risk and survival at individual level, Nomogram model was established using the genes with significant transcriptional change. Figure 7 shows the details of Nomogram model. Gene scores can be calculated as described in the methods. Values for linear predictor can then be calculated with gene scores, and the corresponding survival probability (risk) can be determined at various time points. As a result of the Nomogram model, an eight‐gene signature‐based risk score formula was established as: Risk Score = 0.137*SPRR2D + 0.2294*NEUROD1 ‐ 0.49489*FGF18 + 0.30546*PLAC1 + 0.23379*SAA2 ‐ 0.38038*DSCAML1 + 0.14451*HMX3 + 0.68936*OTOP3, in which genes names represent gene scores for indicated genes.

## 4 DISCUSSION

In this study, we obtained WES and RNA‐seq data from primary cancer tissues of stage IV CRC and correlated the data with patient clincopathologicial and prognosis information. Multivariate model identified cancer location (left or right) and RNA levels of several genes as the independent risk factors for patient prognosis. This was the first time that RNA level changes were identified as prognostic factors for stage IV patients who underwent surgery on primary cancer and systematic therapy on liver metastasis. Furthermore, individual patient survival can be predicted by the Nomogram model we established, enabling the assessment of patient prognosis before any therapy. Our study provided objective tools for prognosis prediction for the subgroup of stage IV CRC patients, and facilitated the early assessment of patient condition along with other clinicopathological factors.

Previous investigations on the prognosis stratification of stage IV CRC have mainly focused on four types of influencing factors, including clinicopathological factors, genetic alterations, transcriptional alterations, and epigenetic changes.^7^, ^14^ Clinicopathological factors mainly involve age, sex, BMI, race, T, N, M staging, tumor location, pathological subtype, tumor grade, smoking history, family history, etc. Genetic alterations include germline gene mutations, somatic mutations, copy number variations (CNVs), tumor mutational burden (TMB), and microsatellite instability (MSI) status, etc. Transcriptional alterations involve changes of mRNA, miRNA, or lncRNA levels. Epigenetic alterations mainly include methylation and hydroxymethylation alterations.

Clinicopathological factors were widely reported to influence the prognosis of stage IV patients. Left‐sided CRC was shown to have better prognosis than right‐sided CRC by several studies.^15^, ^16^ Our present study also confirmed the findings in the specific group of stage IV CRC patients. T staging was found as the independent risk factor for CRC, and lower T staging correlated with significantly longer survival time and better prognosis.^17^ Clinical staging (stage I‐IV) was also an influencing factor of CRC prognosis,^17^ but it correlates with T, N, and M staging closely and may not be an independent risk factor.^17^ Highly differentiated CRC generally exhibited better prognosis than moderately and low‐differentiated CRC,^18^, ^19^ while mucinous adenocarcinoma generally exhibited poorer prognosis than tubular adenocarcinoma.^18^, ^19^ Smoking was shown as a risk factor for CRC, and familial history of colorectal tumors and inflammatory bowel disease, especially history of hereditary cancer, was also regarded as a risk factor for CRC.^18^, ^19^, ^20^ Unhealthy diet habit and life style was also shown to be risk factors of CRC.^18^, ^19^, ^20^

Genetic alterations were also proved to be prognostic for stage IV CRC patients. KRAS gene is among the most widely investigated genes in CRC. It has been reported that patients with wild type KRAS exhibited significantly better prognosis than those with mutant KRAS for stage IV patients.^21^ Indeed, target therapy with cetuximab and panitumumab is sensitive for KRAS wild type patients, while not suitable for those with KRAS mutations, and it is not surprising that the patients with wild type KRAS benefited from TKI therapy and exhibited better survival.^9^ It was also reported that BRAF mutations were not only associated with poor prognosis, but also linked with less benefit when treated with anti‐EGFR antibodies in metastatic colorectal cancer (mCRC).^22^ dMMR/MSI‐H mCRC was shown to have a poor prognosis following conventional chemotherapy, but exhibited good prognosis following immunotherapy by many studies.^23^ More recently, TMB appeared as a new marker for stratifying patients with immunotherapy, in which patients with high TMB exhibited better response than those with low TMB.^24^ CRC patients with germline mutations (such as Lynch syndrome) represent a specific population of high risk hereditary cancers with MSI‐H/high TMB/dMMR.^25^, ^26^ Patients with germline mutations generally exhibit younger age of cancer onset, earlier occurrence of metastasis, multiple metastasis, and rapid development of the disease than sporadic CRC patients. Immunotherapy may have better control of hereditary CRC than other systematic strategies.^25^, ^26^

In contrast to genetic alterations at DNA level, transcriptional alterations, and expression alterations showed a large variety of observations with little consensus. For example, some studies identified the expression of single markers for prognosis prediction, such as CD133,^27^ HSF4,^28^ and PLAC1,^29^ etc., while other studies identified panels of genes with significant efficacy in prognosis prediction at protein expression levels, mRNA levels or miRNA levels of multiple genes.^30^, ^31^, ^32^, ^33^ Almost no overlapping of genes was found among the studies. The reason for the huge discrepancies in these studies may involve the large variation of gene transcription and expression profiles across different populations or individuals, the method of tissue sampling, the method for sample treatment and measurement, and the heterogeneity of CRC.

In this study, we showed no stratification by genetic alterations, which was different to previous observations. The reasons for this discrepancy may involve the following aspects. First, the stratification by KRAS and BRAF mutations may not be present in this specific subpopulation, as the patients involved in this study received surgery of the primary cancer without removing the metastasis. The specific situation and therapeutic strategy may mask the stratification by mutations. Second, the number of patients in this subpopulation were still low, leading to the limited number of patients with mutations, which may influence the significance of statistics. Third, the rapid optimized chemotherapy and best supportive care may prolong the survival of patients previously with poor prognosis, and masked the stratification by mutations.

In order to investigate the stratification factors for the subpopulation, we identified differentially transcripted genes and relevant functions in this subpopulation, and established the stratification model by transcriptional alterations focusing on the differential mRNA levels, with specific focus on aberrant pathways (such as Notch and Wnt etc.) and functions (such as ion channel, transporter, and receptor function). Based the above findings, univariate and multivariate Cox regression identified genes significantly correlated with patient prognosis, and stratification model was established. Since the prediction of prognosis in a population cannot be applied to individuals by Cox regression, Nomogram model was subsequently established by combining all significant genes from Cox regression, which facilitated the prognosis prediction at individual level using the mRNA data from RNA‐seq.

Using the primary cancer tissue, an early assessment can be done to provide a prediction before any therapy. The method is clinically applicable as primary cancer tissue can be obtained from colonoscopy or surgery, and RNA‐seq test is easily accessible in terms of both experiment methods and costs. Fresh tissues or frozen tissues are needed to perform the RNA‐seq test, while FFPE samples are not ideal for RNA‐seq. This may not be an obstacle, as collection of fresh or frozen tissues is becoming a routine in CRC examinations. Due to the rapid development of gene techniques, it is possible that the genetic test‐based models may play more roles in future diagnosis and prognosis prediction.

Patients who performed radical surgery on primary tumor but did not perform the resection of liver metastases represent a subpopulation of stage IV CRC, which has been rarely studied. These patients had their primary cancer removed but their liver metastases were assessed as unresectable, and therefore, they generally received chemotherapy as the subsequent treatment for liver metastasis, including FOLFOX or FOLFIRI+targeted therapy.^3^, ^4^ In the selected cohort in this study, no patients received liver metastasis removal afterwards, which provided us an opportunity to study the long‐term prognosis of these patients. It appeared that the prognosis of this subpopulation was better than the stage IV CRC patients without any surgical opportunity.^34^ These patients generally have unresectable multiple primary lesions and metastases, and require systematic therapy. It also appeared that the prognosis of this subpopulation in this study was worse than the stage IV CRC patients with both primary lesion and metastasis removed, rationalizing the therapeutic strategy that the removal of both primary and metastatic lesions should be performed whenever possible to enhance the opportunity of radical treatment, which showed better prognosis.^13^

The present study had some limitations. First, the models established in this study were based on a relatively small number of patients and its effectiveness needed larger sample size to verify. Second, future prospective validation of the models is needed as the current study is retrospective. Third, the stratification of key mutations still needs further examination in this population. Fourth, model from one omics (here it refers to the model established by RNA data) may have bias and therefore combined model from multiple omics with clinical and demographic information is needed to improve the capability of models.

## 5 CONCLUSIONS

The prognostic factors for stage IV CRC patients who underwent radical surgery on primary cancer and systematic therapy on liver metastasis were identified for the first time. Location (left or right) and the RNA levels of several genes were identified as the independent risk factors for the subgroup of patients. Nomogram model was established to predict individual patient prognosis. Future assessment of patient prognosis can be performed with the model along with clinicopathological factors.

## CONFLICT OF INTEREST

All authors claim no conflict of interest.

## AUTHOR CONTRIBUTION

Bian Wu, Jinwei Yang, Jingyi Shao, and Yun Shang designed the study. Bian Wu, Jinwei Yang, Zhiwei Qin, Hongping Yang, Jingyi Shao, and Yun Shang collected the samples and clinical information and diagnostic information. Bian Wu, Jinwei Yang, Zhiwei Qin, and Hongping Yang sent the samples for sequencing experiments and analyzed the sequencing data. All authors contributed to data analysis, drafting or revising the article, gave final approval of the version to be published, agreed to the submitted journal, and agree to be accountable for all aspects of the work. Bian Wu, Jinwei Yang, Jingyi Shao, and Yun Shang proof read the manuscript. Yun Shang submitted the manuscript.

## FUNDING INFORMATION

This study was supported by the Applied Basic Research Joint Special Project of Yunnan Provincial Science and Technology Department and Kunming Medical University [2018FE001‐(110)]. All funders did not participate in the study design, study implementation, data collection, data analysis, data interpretation, and manuscript writing of the study.

## ETHICS APPROVAL AND CONSENT TO PARTICIPATE AND PUBLICATION

This study was approved by the ethnic committee of the First People's Hospital of Yunnan Province. Written informed consent was waived as this was a retrospective study. Consent for publication was waived as this was a retrospective study.

## Supporting information


Table S1

Table S2

## Data Availability

The datasets generated and/or analyzed during the current study are available from the corresponding author upon reasonable request.
